# Ferritin Vaccine Platform for Animal and Zoonotic Viruses

**DOI:** 10.3390/vaccines12101112

**Published:** 2024-09-27

**Authors:** Sohrab Ahmadivand, Robert Fux, Dušan Palić

**Affiliations:** 1 Faculty of Veterinary Medicine, Ludwig-Maximilians-Universität München, 80539 Munich, Germany; 2 Institute for Infectious Diseases and Zoonoses, Ludwig-Maximilians-Universität München, 80539 Munich, Germany; robert.fux@lmu.de

**Keywords:** viruses, vaccines, veterinary, ferritin, self-assembling protein, zoonoses

## Abstract

Viral infections in animals continue to pose a significant challenge, affecting livestock health, welfare, and food safety, and, in the case of zoonotic viruses, threatening global public health. The control of viral diseases currently relies on conventional approaches such as inactivated or attenuated vaccines produced via platforms with inherent limitations. Self-assembling ferritin nanocages represent a novel vaccine platform that has been utilized for several viruses, some of which are currently undergoing human clinical trials. Experimental evidence also supports the potential of this platform for developing commercial vaccines for veterinary viruses. In addition to improved stability and immunogenicity, ferritin-based vaccines are safe and DIVA-compatible, and can be rapidly deployed in response to emerging epidemics or pandemics. This review discusses the structural and functional properties of ferritin proteins, followed by an overview of the design and production of ferritin-based vaccines, the mechanisms of immune responses, and their applications in developing vaccines against animal and zoonotic viruses.

## 1. Introduction

Infectious diseases remain a major challenge affecting animal health and food safety, with viral diseases accounting for a significant proportion of cases [1]. Viruses are responsible for approximately half of the World Organization for Animal Health (WOAH) notifiable terrestrial and aquatic animal diseases, originating from 22 different families [2,3].

Several of these viral diseases can also be transmitted to humans (zoonotic diseases), posing a risk of infection through direct or indirect contact. Notably, around 60% of diseases in humans can be traced back to animal origins, leading to high mortality rates and the potential for adaptation to humans, resulting in epidemics and pandemics, such as those caused by avian influenza viruses [4,5].

Prevention through vaccination is regarded as the most successful strategy against animal viral diseases, especially zoonoses. Currently, three main types of vaccines, namely inactivated vaccines, live attenuated vaccines, and recombinant subunit vaccines are widely used in the veterinary field [1]. However, safety concerns persist, and the viral antigenic proteins are subject to amino acid substitutions, following mutation in their corresponding genes. For instance, live attenuated viruses like the globally marketed Classical Swine Fever Virus (CSFV) has failed to meet serological criteria for differentiating infected from vaccinated animals (DIVA) [6]. Additionally, the reliance on chicken embryos for development and the low antibody levels associated with inactivated influenza vaccines highlight the limitations of commercial whole-virion vaccines, contributing to the emergence of new virus strains and ongoing outbreaks even among vaccinated animals [7].

Nucleic acid-based vaccines, considered third-generation vaccines, have several drawbacks, despite their promising results. These include the high manufacturing costs of mRNA vaccines and their low thermostability, which necessitates ultra-freezers. Additionally, regulatory compliance for DNA vaccines is hindered by safety concerns related to genetically modified organisms (GMOs). This has become a significant barrier to the approval of the APEX-IHN^®^ DNA vaccine for fish in Europe, for example [8,9].

As a result, there is a concerted effort to develop more effective and stable subunit vaccines that are safer and widely used in veterinary medicine [1]. A novel approach involves displaying structurally defined antigenic proteins in high copy numbers on the surface of self-assembling nanocages. Unlike virus-like particles (VLPs), these nanocages can serve as an ideal scaffold for enveloped viruses, substituting lipid membranes and matrix proteins [10,11].

Ferritin is a major intracellular iron storage protein found in most living organisms. It consists of 24 identical subunits that spontaneously self-assemble, forming nanoparticle complexes with internal and external diameters of 8 and 12 nm, respectively [12]. Self-assembling ferritin nanoparticles (NPs) are biodegradable and biocompatible, exhibiting remarkable thermal and pH stability, as well as reversible spontaneous assembly/disassembly, which enables mucosal and oral delivery [10,12]. The size and unique structure of ferritin allow for the conjugation and multiple display of viral antigens/epitopes, enhancing uptake and delivery by antigen-presenting cells, directly activating B cells, and increasing cross-linking of B-cell receptors (BCRs). This results in robust and durable antibody production [13,14,15]. Furthermore, ferritin can enhance the innate immune system through the activation of Pattern Recognition Receptors (PRRs) [10,16].

Ferritin NPs have been utilized to develop vaccines against various viruses in both prokaryotic and eukaryotic expression systems, primarily for human use. Some of these vaccines have entered clinical trials for influenza, HIV, EBV, and SARS-CoV-2 [16,17]. Experimental evidence also supports the potential of the ferritin platform for developing safe DIVA vaccines against WOAH-notifiable and emerging livestock and zoonotic viruses, as well as viral pathogens in aquaculture. These vaccines provide strain cross-protection and elicit greater humoral and cellular immunity compared to commercially available inactivated or subunit vaccines [10,17,18,19,20,21,22,23].

This review discusses the structural and functional properties of ferritin, followed by an overview of the design and production of ferritin-based vaccines, mechanisms of immune response, and applications in developing vaccines for animal and zoonotic viruses.

## 2. Ferritin Platform

### 2.1. Structures and Characteristics

Ferritin is a major intracellular iron storage protein which consists of a heavy chain (21 kD) and a light chain (19 kD) in mammals, while teleost fish have also a medium chain (20 kD). In contrast, the proteins found in bacteria and plants contain only one subunit resembling the vertebrate heavy chain [12,13,14,15,16,17,18,19,20,21,22,23,24]. Despite variations in amino acid sequences (up to 80%) and the presence of heme moieties in some bacterial ferritins, ferritins from different species and even mammalian heavy and light chains, which share only about 55% sequence similarity, exhibit similar structures [25].

**Figure 1:**
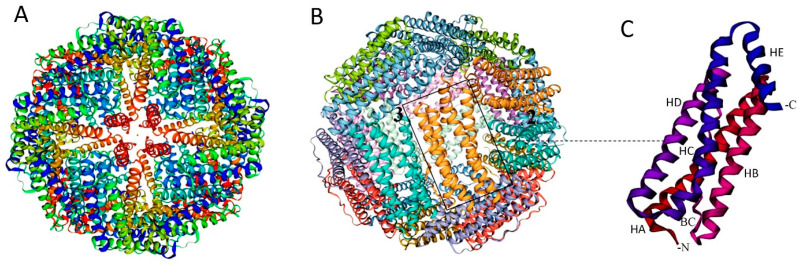
Structure of ferritin nanocage. Schematic view of the ferritin nanocage with four (**A**) and two and three (**B**) axes of symmetry. (**C**) Ferritin monomer featuring the five α-helices (A, B, C, D, and short E α-helix). BC: Protein loop connecting B to C α-helix. Created with MOLSCRIPT [30].

The protein comprises 24 identical subunits with a mass of approximately 500 kDa that assemble in octahedral (234) symmetry to form ion channels (6 hydrophobic four-fold channels, 8 hydrophilic three-fold channels, and 12 two-fold axis channels) and nanocage complexes (75 Å diameter) with inner and outer diameters of 8 and 12 nm, respectively [12,24] (Figure 1A,B). This structure allows for the storage of up to 4500 iron atoms in the form of ferric oxyhydroxide (FeOOH), combined with varying amounts of phosphate [25]. There are also distinct groups within the ferritin superfamily, such as archaeoferritin and DNA-binding proteins (Dps) from starved cells, which consist of only 12 subunits or are monomeric proteins [26].

Ferritin subunits consist of a four-α-helix bundle (A, B, C, and D helices) along with a short fifth E-α-helix at the C-terminus, connected by four flexible chains that run from the outside to the inside. This arrangement places the C-terminal end of the ferritin chain inside the assembled nanoparticle [10,24] (Figure 1C). The B-C loop, which is the longest, at 19 amino acids, connects the C-terminal end of the B-helix to the N-terminal end of the C-helix. The C-terminal, along with the B- and D-helices, faces the inner side of the ferritin nanocage, while the N-terminal, L loop, and A- and C-helices are accessible to the solvent [27].

Although the overall mechanism of ferritin self-assembly is not yet fully understood, it is believed to occur through subunit–subunit interactions. Initially, two single subunits form a dimer, which then assembles into a tetramer and subsequently a hexamer, ultimately resulting in a complete protein cage. The interactions between monomers and dimers may also be involved in the reversible reassembly process of ferritin [28].

The internal cavity of ferritin allows for the incorporation of various cargoes, making it ideal for drug delivery [28]. Additionally, the external surface can be easily modified for antigen or hapten presentation via the N-terminus. Mutational studies have also indicated that the C-terminal region plays a significant role in ferritin stability and assembly capacity [29].

Ferritin exhibits remarkable thermal and pH stability, due to its unique structure and the numerous salt bridges and hydrogen bonds formed between subunits. It can withstand temperatures of up to 80–100 °C and pH ranges of 3 to 10 [24]. Ferritin from the hyperthermophilic archaeal anaerobe (*Pyrococcus furiosus*) can endure incubation at 100 °C for one day, or autoclaving at 120 °C for 30 min without loss of activity [31]. The ferritin protein shell begins to disassemble at pH levels below 3.4; however, this process is reversible when pH levels are subsequently elevated, making this platform an excellent candidate for mucosal applications and oral delivery [10].

Protein stability is believed to be influenced by conjugation methods. For instance, chemically cross-linking antigenic proteins to virus-like particles has been shown to enhance resistance to low pH, proteases, and mechanical stress. This should also be considered in the development of ferritin-based vaccines [32]. While exogenous proteins are expected to reflect modifications and size variations of the cages, our recent study [10] demonstrated excellent stability and similar tolerance patterns between the fusion protein and ferritin nanoparticles. Both were highly stable after three weeks of storage at 4 °C and during −80 °C freeze–thaw cycles. They also maintained stability at pH 8.0 and pH 3.0 at 15 °C for up to four hours, mimicking gastrointestinal conditions [10].

The use of ferritin as a carrier for drug delivery and vaccine development has reported almost no toxic side effects. Nevertheless, it is essential to evaluate the potential toxicity of ferritin-based vaccines at higher doses to ensure their safety. Assessment models have been employed in toxicological studies of ferritin NPs. For instance, Charlton et al. [33] evaluated the toxicity of horse spleen ferritin in an in vivo study. Their findings indicated that ferritin NPs, administered at doses intended for targeted MRI molecular imaging, caused no short-term mortality or morbidity and had no significant effect on the structure or function of vital organs in the mice. Additionally, a recent in vitro study [10] using MTT viability assays on infectious hematopoietic necrosis virus (IHNV) ferritin-based vaccines and ferritin NPs in zebrafish liver (ZFL) cells showed no cytotoxicity up to 100 µg/mL for 14 h. This indicates that the NPs are biocompatible with living cells at very high levels, making them valuable not only for aquatic animals but also for mammalian and human vaccines and biomedical applications.

### 2.2. Design and Production

#### 2.2.1. Antigen Display on Ferritin Scaffold

The display of antigens on the outer surface of protein nanoparticles has primarily been demonstrated using three approaches: genetic fusion, chemical and enzyme-mediated conjugation, and the SpyTag/SpyCatcher system [34].

##### Genetic Fusion

**Figure 2:**
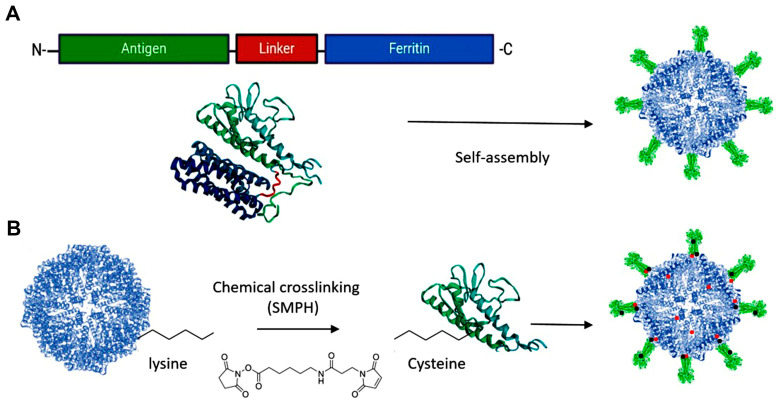
Design Approaches for Antigen Presentation on Ferritin Platforms. (**A**) Genetic fusion: the gene encoding an antigen of interest is fused to the outer surface of the ferritin platform. (**B**) Chemical cross-linking: side chains on the antigen and the platform are connected by a cross-linker, e.g., SMPH [succinimidyl 6-(β-maleimidopropionamido) hexanoate]. Created with Biorender.com (accessed on 23 September 2024).

Antigen subunits can be genetically fused to either the C- or N-terminus of proteins. However, increasing structural complexity and size of the antigen can compromise NP assembly and integrity [34,35]. The inner surface of the ferritin nanocage, accessible via the C-terminus, can be utilized for drug delivery, while the N-terminus is located on the nanoparticle’s surface, making it accessible for genetic fusion of antigens in vaccine design (Figure 1C and Figure 2A).

Genetic fusion of antigenic proteins, typically using Gly-Ser flexible linkers on the outer surface of ferritin, has been widely employed for developing vaccines against animal and zoonotic viruses, ranging from simple peptides to complex trimers (Table 1). A notable example is the influenza vaccine currently in human clinical trials, which has been developed by genetically fusing hemagglutinin (HA) to ferritin [36]. Importantly, genetic fusion of short fragments to a ferritin platform may enable the production of soluble, complex glycoproteins even in prokaryotic systems [10,36].

##### Chemical and Enzyme-Mediated Conjugation

Chemical conjugation offers a distinct advantage over other techniques by enabling the attachment of multiple antigens to monomeric proteins [37]. Although chemical methods can be more complex and require specific conditions and reagents compared to genetic fusion, this complexity can result in heterogeneous decoration of nanoparticles, potentially affecting their presentation to the immune system [34]. Despite these challenges, chemical conjugation has been demonstrated to significantly improve immune responses [38]. Among these methods, chemical cross-linking is the most commonly used technique to facilitate the binding of antigens to native self-assembling platforms. Examples include commercial vaccines like M2-HPV (Merck Co., Rahway, NJ, USA), influenza A (Cytos Biotechnology AG, Schlieren, Switzerland), and SARS-CoV-2 (Saiba-AG, Pfäffikon, Switzerland), which utilize chemically cross-linked VLPs [39,40].

Chemical cross-linkers consist of natural or synthetic molecules that create covalent bonds between proteins. Proteins and peptides contain primary amines (-NH2) at their N-terminus and in the side chains of lysine residues, as well as sulfhydryl (-SH) groups in the side chains of cysteine residues, which can be targeted for conjugation with antigenic proteins on ferritin [34] (Figure 2B).

However, proteins often have multiple primary amines and hydroxyl groups available on their surfaces, which can lead to variability and unpredictability in the outcomes of chemical cross-linking [35]. Additionally, cysteine residues introduced into genetically engineered proteins for site-specific attachment might disrupt existing disulfide bonds, posing another limitation to this approach [34,35].

Click chemistry presents an alternative technique for rapid and efficient covalent conjugation and coupling of proteins to a protein platform or VLPs through the incorporation of unnatural amino acids (uAAs). The conjugated protein must then be functionalized with a ligand that has an affinity for the uAA. A common click-chemistry reaction involves substituting an amino acid in the antigen (methionine) with azide–alkyne analogues in the presence of Cu(I) catalysis for conjugation [41].

Chemically Induced Dimerization (CID) is another conjugation method by which antigenic proteins and peptides can bind to the ferritin platform in the presence of specific small molecules, enzymes, or other dimerizing agents. In contrast to bifunctional cross-linking agents used in chemical conjugation, CID provides superior affinity, specificity, and faster reaction kinetics. Similar to genetic fusion, CID ensures product homogeneity. However, it limits conjugation to the N- or C-terminus of proteins, as the peptides interacting with dimerizers, such as FK506 binding proteins (FKBPs), must be attached at these termini [34].

Although the process is fast, selective, and results in high yields, its complexity and cost with side reactions of the unnatural amino acid are barriers to scale-up [34,35]. Instead of a dimerizer, the reaction can be mediated by a catalyst (i.e., enzyme) that does not restrict the binding of a protein to either the C- or N-terminal of ferritin, as seen in other approaches [34,42].

##### SpyTag/Catcher Design

**Figure 3:**
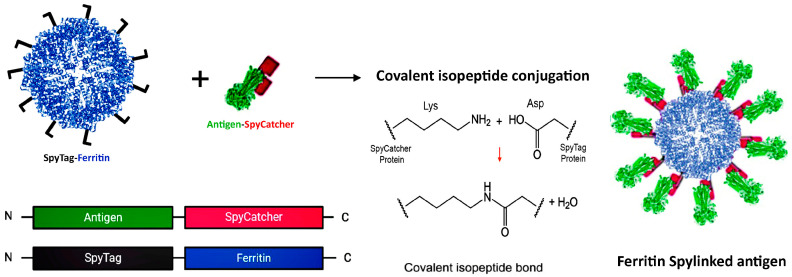
SpyTag–SpyCatcher Design for Antigen Presentation on the Ferritin Platform. A SpyTag fused to the N-terminus of ferritin and the antigen–SpyCatcher fusion are expressed separately (**left panel**). Upon mixing, the SpyCatcher/SpyTag side chains form a covalent isopeptide bond with the release of water (**middle panel**), resulting in decorated vaccine nanoparticles via antigen display on the surface of the ferritin scaffold (**right panel**). Created with Biorender.com (accessed on 23 September 2024).

The SpyTag–SpyCatcher system is a novel method for vaccine development that enables efficient attachment of antigens to protein platforms while preserving their native structure. Regardless of antigen size, it allows for independent production and coupling of components under standard conditions [43]. In this system, a split variant of the fibronectin-binding protein from *Streptococcus pyogenes* is utilized, which comprises SpyCatcher (113 amino acids) and SpyTag (13 amino acids) [43,44]. Platform nanoparticles conjugated with SpyCatcher can form a stable isopeptide bond with SpyTag attached to the antigen, or vice versa (see Figure 3). This reaction can occur at either the N- or C-terminus, since it is mediated through side chains [43]. Studies have shown that displaying viral antigens on self-assembling protein NPs using SpyTag/SpyCatcher elicits stronger immunization than direct genetic fusion produced in eukaryotic expression systems [45].

This system has been successfully applied to infectious viruses in animals, such as displaying the E2 protein of Classical Swine Fever Virus [46] and the dimer receptor-binding domain (RBD) of the Porcine Deltacoronavirus (PDCoV) spike protein [47] on the ferritin scaffold.

The SnoopCatcher–SnoopTag system can also be considered for antigen display on ferritin nanoparticles, where the reaction results in covalent binding and the release of ammonia instead of water [43].

#### 2.2.2. Expression and Purification

##### Expression Systems

For vaccine development, especially against emerging and zoonotic viruses, various expression systems for ferritin from different sources such as Archaeoglobus fulgidus, Helicobacter pylori, Trichoplusia ni, *E. coli*, and Pyrococcus furiosus have been investigated [48]. Among them, *H. pylori* ferritin NPs with a three-fold axis of symmetry are particularly interesting and are increasingly used for many human and veterinary vaccines. This is likely due to their lower sequence homology and limited iron content when heterologously produced, which minimizes the risk of autoimmune reactions [36].

Self-assembling ferritins have been produced in various hosts, including bacteria (*E. coli*), insects (Sf9, High-Five, and Drosophila S2 cells), and mammalian cells (human embryonic kidney (HEK) and Chinese hamster ovary (CHO) cells) [12,24,28].

Baculovirus systems exemplify a method for producing high levels of recombinant proteins while providing appropriate post-translational modifications. This approach offers numerous advantages, including manufacturing speed, flexible product design, inherent safety, scalability, and improved technologies such as Bac-to-Bac and FlashBAC™ systems. These advancements have recently been applied to ferritin-based viral vaccines like FMDV and PDCoV [22,47].

However, the mammalian expression system is less commonly used for animal vaccines compared to human vaccines. Recently, a CSFV-ferritin vaccine produced by CHO cell lines induced greater immunity without pathological lesions in challenged pigs, comparable to the subunit vaccine produced by the baculovirus expression system [46].

Mass production of such vaccines using a rapid and inexpensive expression system (*E. coli*) has further streamlined the manufacturing process. Most ferritin-based vaccine candidates against animal and zoonotic viruses, such as Canine Distemper virus (CDV), PDCoV, IHNV, Nipah virus (NiV), MERS-CoV, Zika virus, and influenza virus [36] that have entered clinical trials have been produced using this prokaryotic expression system. 

CDV hemagglutinin (HA) ferritin expressed in *E. coli* induced immunity comparable to that of full-length eukaryotic expression (DNA vaccine) [18]. In vivo investigations have also shown that *E. coli*-expressed Porcine reproductive and respiratory syndrome virus (PRRSV) GP5-ferritin can effectively resist infection by highly pathogenic strains and significantly improve piglet survival compared to a commercially available inactivated vaccine [20]. Additionally, we recently demonstrated the development of a highly stable and soluble recombinant glycoprotein of IHNV with antiviral characteristics using the ferritin scaffold in the E. coli system, whereas the antigenic protein alone was insoluble [10].

Similar to other bacterial self-assembling protein nanoparticles, ferritin requires limited post-translational modifications, and can be used as a novel vehicle to rescue and produce soluble proteins that are otherwise difficult to obtain using conventional methods. However, some modifications, such as incorporating an N-linked glycosylation site, may be necessary to facilitate the expression of complex glycoproteins.

The conjugation system is an important factor affecting the yield, immunogenicity, and stability of the nanoparticles [34,35], which should be considered, alongside the optimal conditions for culturing the expressing cells.

##### Purification and Self-Assembly

Purification of the protein nanoparticles is often performed using various chromatographic methods, such as ion exchange, lectin affinity, or metal affinity, followed by size exclusion chromatography (SEC), requiring customization for each potential vaccine. Considering the location and number of histidine residues, the polyhistidine (His) tag via IMAC Ni^2+^ resins is commonly used for successful affinity purification of ferritin fusions. Due to surface exposure, the N-terminus of ferritin is preferred for His-tag insertion. The installation of a polyhistidine tag with 6–8 histidine residues into the flexible loops on the surface of SARS-CoV-2 spike-modified ferritin has been shown to facilitate purification; however, with four or five histidine residues, the protein was not purified [48]. Other tags, such as Strep-tag^®^ for mammalian expression of ferritin vaccine nanoparticles, have also been used to facilitate purification [49].

The stability and self-assembly of ferritins are significantly influenced by ionic strength, with higher NaCl concentrations leading to increased aggregation [50]. Research indicates that ferritin can be released in a soluble form from *E. coli* at lower NaCl levels (<50 mmol/L) [44]. To improve the quality of the protein NPS, it is crucial to adjust Fe^2+^ concentrations during expressing and the purification process [51]. Furthermore, using a flexible and sufficiently long linker can ensure steric assembly for multimeric nanoparticles [51]. The ferritin protein shell tends to disassemble under highly acidic or basic conditions, but this disassembly is reversible when the pH is adjusted to neutral values [10].

Overall, optimal concentrations of Fe^2+^ ions, salt, pH, and fusion linkers are among the factors contributing to proper self-assembly and should be carefully considered when designing ferritin-based vaccines.

### 2.3. Immune Response Mechanisms of Ferritin-Based Vaccines

**Figure 4:**
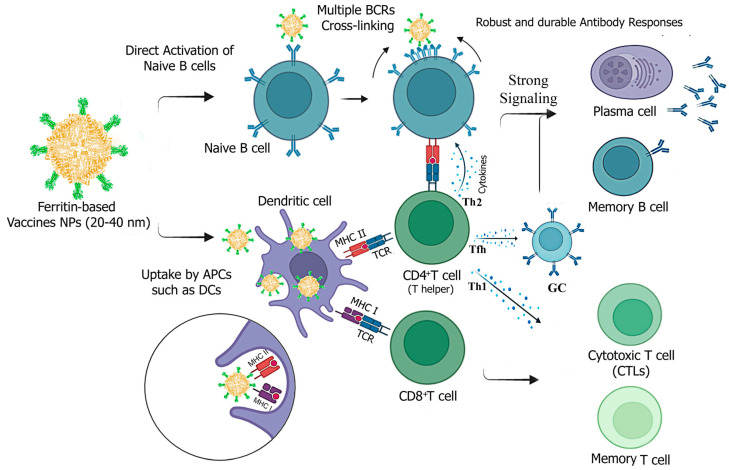
Potential mechanisms of immune responses induced by ferritin-based vaccine NPs against infectious viral diseases. Ferritin-vaccine NPs are processed and presented by both MHC-I and MHC-II for recognition by CD8+ and CD4+ T cells with T cell receptors (TCRs). They enhance immunity mainly through direct B-cell activation and improved trafficking by APCs. The vaccine NPs can induce humoral immunity by directly activating B cells through cross-linking of BCRs and by MHC II-mediated activation of CD4+ T helper cells. T helper 2 (Th2) cells secrete cytokines like IL-4, whereas T follicular helper (Tfh) cells produce essential cytokines such as IL-21, which directly support B-cell activation and germinal center responses. This results in strong signaling that induces robust and durable antibody secretion by plasma cells and the generation of memory B cells. To elicit cellular immune responses, immature CD8+ T lymphocytes proliferate and differentiate into cytotoxic T cells (effector) and specific memory CTLs. CD4+ T helper cells (Th1) can also support activated CTLs by secreting cytokines (e.g., IFN- γ). Created with Biorender.com (accessed on 23 September 2024).

There is increasing evidence that ferritin-based vaccines induce stronger cellular and more robust, durable humoral immune responses compared to conventional subunit vaccines (Figure 4). This is primarily due to the unique structure of nanoparticles, which enhances stability, cellular uptake, and recognition by the immune system, leading to the desired immune responses.

Vaccine particles ranging from 5 to 100 nm can be directly delivered to lymph nodes (LNs) and interact with B cells. In contrast, particles up to 500 nm in size need to be taken up by antigen-presenting cells (APCs), such as migratory dendritic cells (DCs), to transport antigens to LNs, where they are then recognized and processed for presentation [52,53].

Ferritin-based vaccine candidates typically range from 20 to 40 nm in diameter after self-assembly, making them smaller than many NPs used in vaccine development [10,49,51]. This small size facilitates movement across membranes, enhancing antibody production through increased nanoparticle trafficking to lymph nodes in association with DCs and direct activation of B cells via improved antigen delivery [52,53]. Consistently, ferritin-based influenza [13], SARS-CoV-2 [16], and HIV [54] vaccine NPs in this size range have shown increased trafficking and deposition in lymph nodes.

The surface structure and spherical shape of ferritin NPs mimic viral structures and resemble pathogen-associated molecular patterns (PAMPs) recognized by pattern recognition receptors (PRRs). This resemblance can enhance cellular uptake and induce innate immunity upon binding to APCs [15]. Notably, robust innate antiviral immunity has been observed in fish macrophage cells following IHNV ferritin vaccination [10].

Although typically negatively charged, the surface charge of ferritin can vary depending on the subunit composition, allowing for greater flexibility in targeting ferritin molecules across different membranes [24]. For Zika and IHN viral vaccines, no significant change in NP charge has been observed when antigenic proteins are displayed on the surface of ferritin [10,23], though it may depend on the charge of the fusions. Negatively charged nanoparticles can also be effectively taken up by antigen-presenting cells, with those smaller than 100 nm being particularly efficient due to their reduced interaction with positively charged non-immune cells [53].

Recent studies on various viruses, including IHNV, FMDV, Influenza, CSV, and CDV, indicate that ferritin alone typically does not elicit significant humoral or cellular immune responses, even at higher doses [10,18,19,21,22]. These findings suggest that the immunogenicity and antiviral activity primarily depend on the attached antigen, rather than on ferritin itself. Nonetheless, it remains essential to monitor immune responses and assess potential interactions between anti-ferritin antibodies and vaccine efficacy to ensure the effectiveness of multi-dose vaccination strategies.

High levels of neutralizing antibodies depend on the efficient recognition and cross-linking of multiple neutralizing epitopes with B-cell receptors. The engagement of multimeric BCRs through direct interactions between intact nanoparticles and B cells contributes to the potent activation of B cells and the subsequent differentiation of germinal centers [13].

The unique structure of ferritin, composed of 24 subunits and featuring repetitive and organized structures on its surface, allows for the multiple display of viral antigens/epitopes. This enhances the cross-linking of BCRs and promotes antibody production. Influenza HA-ferritin vaccine NPs have been shown to strongly stimulate cross-linking of BCRs in vitro using engineered B-cell lines expressing HA-specific BCRs [14]. This response was not observed with the free HA protein alone [55]. Vaccine NPs interact with major histocompatibility complex (MHC) class I or II molecules, which can directly trigger cellular immune responses or support the development of humoral immunity through interactions between CD4+ T cells and B cells. The processing of exogenous antigens through the MHC II pathway activates CD4+ T cell responses and antibody production. Upon reaching the lymph nodes, DCs process antigens that target CD4+ T cells for T helper cell (Th2) induction. These T helper cells secrete cytokines (e.g., IL-4) that activate B cells through a T cell-dependent pathway and also present directly to naive B cells (direct activation) through T cell-independent pathways following strong signaling from co-receptors (e.g., TLR) or cross-linking of BCRs [37,53].

Induction and persistence of CD4+ and Th2 immune responses, as evidenced by antibody typing and cytokine detection, have been reported with ferritin-based vaccines against viral infections [16,18]. Furthermore, humoral immunity requires interaction between specific B-cell populations and CD4+ T cells, particularly follicular helper T cells (Tfh cells), within the germinal center (GC). This interaction is crucial to generate memory B cells and long-lived plasma cells. Ferritin vaccines for chronic hepatitis B and SARS-CoV-2 have successfully induced efficient Tfh and GC responses [16,56]. Vaccination of mice with influenza HA-ferritin has also shown enhanced and longer-lasting GC responses in draining lymph nodes, focal deposition of antigen in the GCs of these nodes, and increased BCR mutations in memory B cells following HA–ferritin immunization. However, no improvement in HA-specific Tfh cell responses was observed, suggesting that a highly structured and repetitive antigen array may promote germinal center formation through a B-cell intrinsic mechanism, independent of Tfh cell involvement [13].

IL-21 induction, a cytokine primarily produced by T follicular helper CD4+ T cells, has been noted in response to the SARS-CoV-2 spike–ferritin vaccine with the generation of long-lived plasma cells and the production of cross-neutralizing antibodies [57].

While most commercially available vaccines focus on humoral immunity, cellular responses play a crucial role in combating viral infections, particularly in cross-protection. Antigen-presenting cells associated with MHC-I induce cellular immunity via CD8+ T cell responses, which proliferate and differentiate into cytotoxic T lymphocytes (CTLs) and specific memory CTLs to initiate immune responses and directly kill infected cells. CD4+ T helper cells (Th1) can also support activated CTLs by secreting cytokines such as IFN-γ and IL-2 [37,53].

Research has demonstrated that ferritin vaccine NPs targeting animal viruses like PRRSV [20], CDV [18], and CSFV [46], when taken up by dendritic cells, have a high probability of facilitating antigen presentation via MHC-I, thereby eliciting cellular immunity.

## 3. Ferritin-Based Vaccine Candidates for Animal and Zoonotic Viruses

The use of ferritin as a vaccine platform for viruses began in 2013 with H1N1 influenza and has expanded since the SARS-CoV-2 pandemic in 2021, driven by the urgent need for vaccines. Most of these vaccine candidates target human viruses, some of which have entered clinical trials, including those for influenza, HIV, EBV, and SARS-CoV-2 [16,17]. Despite the advantages of this platform, there has been less research and fewer attempts to develop ferritin-based vaccines for veterinary use and other zoonotic viruses. Among these, swine respiratory viruses such as swine influenza, CSFV, PDCoV, and PRRS have seen the most development of ferritin-based vaccines, likely due to their economic importance and the critical role pigs play in interspecies virus transmission, along with lessons learned from the success of SARS-CoV-2 vaccines. Below is an overview of the research progress on the ferritin-based vaccines for animal and zoonotic viruses.

### 3.1. Classical Swine Fever Virus (CSFV)

Classical swine fever virus (CSFV) is an enveloped RNA virus belonging to the family Flaviviridae, and is the causative agent of the only WOAH-listed swine disease in the genus Pestivirus. Although its viral etiology has been recognized since 1903, CSFV continues to pose a significant threat and economic burden to the industry [58].

Vaccination is the primary method for controlling the disease, with the globally commercialized CSFV lapinized vaccine strain C based on a live attenuated virus. However, this approach has failed to meet serologic DIVA criteria. Consequently, safer DIVA-compatible vaccines, such as subunit vaccines, have been explored. Recent studies have identified four antigenic domains (A, B, C, and D) that form two major units, B/C (aa 1–90) and D/A (aa 91–170), in the N-terminal E2 glycoprotein of the virus, encompassing all neutralizing peptides [59]. In this context, the application of a ferritin nanoplatform with soluble E2 antigen has yielded promising vaccine candidates for eradicating CSFV.

Zhao et al. [19] developed a vaccine by fusing the E2 protein of SFV to ferritin nanocages in a baculovirus expression system, which elicited strong immune responses in rabbits, characterized by high levels of neutralizing antibodies and IL-4 and IFN-γ cytokines compared to a conventional subunit vaccine. Additionally, the study noted fewer significant pathological lesions compared to the live attenuated C strain, indicating both safety and promising potential for future vaccine development.

Recently, the superiority of E2 ferritin NPs developed using the SpyTag/SpyCatcher design in a CHO mammalian expression system has been confirmed in lethal-challenge studies with pigs, the primary host for this virus [46]. Therefore, ferritin-based vaccines represent a safe and effective alternative for controlling CSF with DIVA characteristics and could play a significant role in managing and eradicating this disease.

### 3.2. Porcine Reproductive and Respiratory Syndrome (PRRS)

Porcine reproductive and respiratory syndrome virus (PRRSV) is an enveloped, single-stranded RNA virus from the Arteriviridae family, with wo distinct species: *Betaarterivirus europensis* (PRRSV-1) and *Betaarterivirus americense* (PRRSV-2), which share about 60% genetic similarity [60]. PRRSV primarily causes reproductive problems in sows and respiratory disease in piglets, leading to a global pandemic that severely impacts the swine industry [61].

Vaccination is crucial for managing PRRSV spread. After Boehringer Ingelheim introduced the modified live vaccine (MLV) Ingelvac PRRS (strain VR-2332) in 1994, numerous biotech companies followed with their own type 1 and type 2 MLV vaccines or killed vaccines. However, these vaccines have the potential for reversion to virulence, contributing to PRRSV diversity and proving insufficiently effective, particularly against emerging strains [61,62].

In addition to the virus’s immunosuppressive ability and high mutation rate, a significant challenge in controlling PRRSV is its capacity to infect pigs of various ages, leading to diseases characterized by fundamentally different immune responses. This highlights the need for a safer and more effective vaccine that can be administered at any age [62].

The GP5 is a multi-transmembrane protein of PRRSV, essential for inducing neutralizing antibodies, with two key epitopes (GP5IV and GP5B) identified in its extracellular regions [63]. The PRRSFREE subunit vaccine (Reber Genetics Company) is designed to protect against both genotypes of PRRSV, while few monoclonal antibodies can recognize both strains [62].

Ma et al. [64] investigated a vaccine candidate against PRRSV by fusing a modified GP5 protein to ferritin nanocages, utilizing a baculovirus expression system for production. The vaccine demonstrated a robust Th1 cellular immune response and enhanced T lymphocyte activity, with higher levels of neutralizing antibodies compared to inactivated PRRSV vaccines. The study also reported reduced viremia and fewer lung lesions in vaccinated pigs, indicating the vaccine’s potential effectiveness and safety as a promising alternative for PRRSV control.

Another ferritin-based PRRSV vaccine incorporated the B and IV epitopes of the GP5 protein (aa 186–200) into a Plasmodium universal T-cell epitope (PADRE) and this was fused to ferritin nanocages [20]. Compared to an inactivated vaccine in mice, this prokaryote-expressed protein NPs elicited effective humoral and cellular immune responses against PRRSV. This vaccine also resulted in reduced disease duration, milder symptoms, and slower transmission after a lethal challenge, compared to unvaccinated pigs, highlighting its potential as a promising candidate for PRRSV vaccination.

### 3.3. Porcine Deltacoronavirus (PDCoV)

Porcine deltacoronavirus (PDCoV) is a novel swine coronavirus within the Deltacoronavirus genus, characterized by an enveloped, single-stranded RNA genome that causes significant economic losses in the swine industry globally [65].

Since its first report in 2012, several types of vaccines have been evaluated against PDCoV. However, despite its zoonotic potential, no commercial vaccine is currently available. Research on the spike protein subunit expressed in the Bac-to-Bac baculovirus expression system has produced more robust neutralizing antibodies (NAbs) and cellular immune responses than the RBD trimer, lasting up to four months in immunized piglets and resulting in fewer microscopic lesions compared to an inactivated vaccine [66]. To further enhance the development of a safe and effective PDCoV vaccine, ferritin nanocages were used to express the dimer receptor-binding domain of the PDCoV spike protein (RBD-dimer) via SpyTag/SpyCatcher conjugation in a prokaryotic system [47].

The immune responses observed in the homologous prime–boost regimen indicated that the vaccine NPs effectively elicited specific humoral and cellular immune responses in mice. High levels of PDCoV RBD-specific IgG and neutralizing antibodies were detected, similar to those reported by Li et al. [66] for the subunit S protein (approximately 1:26–1:28) in a eukaryotic system, which are sufficient to protect piglets against PDCoV. Additionally, fewer pathological lesions were noted in immunized animals following a lethal challenge with PDCoV. Overall, these results suggest that the ferritin-based vaccine is an ideal candidate for protecting against PDCoV infection and warrants further investigation and optimization.

### 3.4. Influenza A Viruses

Influenza is an acute respiratory infection caused by enveloped, single-stranded RNA viruses from the family Orthomyxoviridae, primarily within the genus Alphainfluenzavirus, which infects humans and various animal species. Influenza A viruses can be classified into more than 130 subtypes based on hemagglutinin (H) and neuraminidase (N) proteins [67].

The main examples of zoonotic influenza viruses are avian influenza subtypes A(H5N1) and A(H9N2) and swine influenza subtypes A(H1N1) and A(H3N2). Commercially available influenza vaccines are mostly inactivated viruses, which have notable drawbacks, including low antibody responses, dependence on chicken embryos for production, and the need to update vaccines to match circulating epidemic strains [68].

The first use of ferritin NPs as a vaccine platform was against Influenza A (H1N1), achieved by fusing hemagglutinin and expressing it in a mammalian system (293F cells) [36]. This vaccine elicited antibody titers more than tenfold greater than those obtained with the commercial inactivated vaccine [36]. Ferritin-based influenza vaccines are being evaluated in Phase I clinical trials (NCT03186781, NCT03814720). Research by Yassine et al. [69] showed a broad range of cross-reactive antibodies by fusing the stem region of the H1HA glycoprotein to ferritin nanocages. The designed vaccine was effective in fully protecting mice from lethal doses of heterosubtypic H5N1 virus, and provided partial protection in ferrets.

The HA–ferritin nanovaccine has demonstrated superior immunity compared to conventional swine influenza virus (SIV) vaccines. Vaccination with the HA extracellular domain of SIV developed using a ferritin platform in a baculovirus expression system has resulted in significantly elevated hemagglutinin inhibition titers, robust antigen-specific IgG production, and increased serum cytokine levels. Notably, the vaccine provided complete protection against lethal H1N1 challenges and significantly reduced the severity of lung lesions caused by the virus in both BALB/c mice and piglets, even when challenged with a heterologous strain exhibiting 81% homology. This underscores its potential as a highly effective candidate for SIV vaccination.

Additionally, cross-protection and improved immunity against H1N1 and H3N2 influenza viruses in mice developed using the same expression system have also been reported [70].

These studies demonstrate that the extracellular hemagglutinin–ferritin vaccine is a promising candidate for SIV vaccination, and that adjuvants such as MF59 and CPG1 may further enhance the humoral and cellular immunity of these vaccines.

### 3.5. Foot-and-Mouth Disease Virus (FMDV)

Foot-and-mouth disease virus (FMDV) is a WOAH-listed, non-enveloped, single-stranded RNA virus from the family Picornaviridae, genus Aphthovirus, which infects cloven-hoofed animals [71]. As with most animal viruses, control of FMDV relies on vaccination. The use of inactivated or attenuated vaccines, however, raises safety concerns and may lead to immunization failures. Chen et al. [22] developed an FMDV vaccine by fusing ferritin to the viral antigen VP1 and the G-H loop using the baculovirus expression system. The vaccine NPs increased FMDV-specific IgG and IgG subclass antibody titers, as well as IL-4 and IFN-γ production and splenocyte proliferation. Additionally, the survival rate of vaccinated mice was improved without pathological lesions. The VP1 subunit vaccine and the VP1–ferritin fusion vaccine provided survival rates of 55.6% and 66.7%, respectively, while the G-H loop ferritin vaccine achieved a survival rate of 77.8% compared to 100% survival in the inactivated vaccine group. Although the partial survival provided by the ferritin–FMD vaccines could be enhanced through optimization of the vaccine design and expression system, these results suggest that the ferritin platform may be more suitable for enveloped RNA viruses than for non-enveloped structures.

**Table 1:** Ferritin-based vaccine candidates against animal and zoonotic viruses.

Virus (Family)	Antigen/ Epitope	Ferritin Source	Expression System	Attachment/ Linkers	Immune Responses	Ref.
CSFV (Flaviviridae)	E2	*H. pylori*	SF9 cells	Genetic	Activation of innate immune cytokines, and neutralizing antibody in immunized rabbits.	[19]
	E2	*H. pylori*	CHO cells	SpyTag/ SpyCatcher	Induction of higher titers of neutralizing antibodies and complete protection of pigs from CSFV challenge.	[46]
PRRSV (Arteriviridae)	Glycoprotein 5 (GP5)	*H. pylori*	SF9 cells	Genetic	Promotion of a Th1-dominant cellular immune response and enhanced specific T-lymphocyte immune responses. Elicited higher neutralizing antibody titers than inactivated PRRSV vaccine in pigs	[64]
	B and IV epitopes (GP5)	*H. pylori*	*E. coli*	Genetic	Immunized mice exhibited the highest neutralizing antibody titers, lymphocyte proliferation index, and IFN-γ levels. Protected piglets against a highly pathogenic PRRSV challenge.	[20]
SIV (Orthomyxoviridae)	Hemagglutinin (HA)	*H. pylori*	SF9 cells	Genetic	Full protection against lethal challenge with H1N1 virus and significantly decreased the severity of virus-associated lung lesions after challenge in mice and piglets. Bivalent H1N1 and H3N2 ferritin vaccine with MF59 and CPG1 adjuvant enhanced cross-immunity and effective protection in mice.	[21,70]
FMDV (Picornaviridae)	VP1 and G-H loop	*H. pylori*	SF9 cells	Genetic	Significantly increased FMDV-specific IgG and IgG subclass antibody titers and cytokines; 66.7% survival rate. but less than inactivated vaccine in mice.	[22]
PDCoV (Coronaviridae)	RBD	*H. pylori*	*E. coli*	SpyTag/ SpyCatcher	Prime-boost regime of dimeric RBD–Ferritin showed specific humoral and cellular immune responses in mice	[47]
CDV (Paramyxoviridae)	Hemagglutinin (HA)- epitopes	*H. pylori*	*E. coli*	Genetic	Induced both Th1 and Th2 immune responses; strong serum titers, and long-term potency of antibodies in serum up to 84 days in mice.	[18]
IHNV (Rhabdoviridae)	Glycoprotein G	*H. pylori*	*E. coli*	Genetic	Induction of robust innate antiviral immunity in vitro in macrophage cells.	[10]
Nipah virus (Paramyxoviridae)	Glycoprotein G	*H. pylori*	*E. coli*	Genetic	Co-displaying of G protein of NiV-M and NiV-B strains on ferritin elicited higher levels of neutralizing antibodies compared to NiV-G subunit and provided complete protection against a lethal challenge with NiV in Syrian hamsters.	[72]
MERS-CoV (Coronaviridae)	RBD	*H. pylori*	*E. coli* (SHuffle^®^ T7)	Genetic	MERS-CoV RBD binding to the cellular receptor hDPP4 was efficiently inhibited by mice sera upon vaccination.	[51]
Zika Virus (Flaviviridae)	E protein domain III	Human ferritin H- chain	*E. coli*	Genetic	Complete protection against the virus infection and eliminated pathological symptoms in the brain of challenged mice.	[23]
Ebola virus (Filoviridae)	Glycoprotein GP	*H. pylori*	Expi-293F cells	Genetic	Elicited potent neutralizing antisera against Ebola virus in mice with cross-neutralizing activity against filoviruses.	[73]

### 3.6. Paramyxoviruses (CDV and NiV)

Paramyxoviruses, belonging to the Paramyxoviridae family, include many animal viruses such as CDV, Newcastle disease virus (NDV), Rinderpest virus, and zoonotic viruses like Hendra and Nipah viruses. There are also some less familiar viruses and novel isolates for which effective vaccines are currently lacking [74].

Canine distemper and the zoonotic Nipah virus (NiV) are enveloped RNA paramyxoviruses for which ferritin-based vaccines are being explored. CDV, a member of the Morbillivirus genus, is highly infectious and causes systemic and often fatal disease in dogs and other terrestrial carnivores [74]. Attenuated strains of CDV are routinely used worldwide as live vaccines to control the virus. However, numerous outbreaks of canine distemper have been reported in vaccinated animals [75]. The hemagglutinin protein is responsible for binding the virus to its receptor and promoting viral fusion, making it the primary target for CDV vaccine research. Wang et al. [18] identified three potential linear epitopes with high hit rates (aa 182–208, 373–420, and 436–471). The combination of these epitopes or YaH4F (aa 436–471), developed using the ferritin platform, resulted in strong cellular immunity with serum titers lasting up to 84 days, comparable to that of a full-length DNA vaccine in immunized mice. Notably, the vaccine NPs were expressed in a prokaryotic system, while the percentage of N-terminal glycosylation of the hemagglutinin protein directly influences the intensity of CDV infection.

Ferritin has also been used to co-display glycoproteins (G) from two different zoonotic Nipah virus strains (NiVM and NiVB) of the genus Henipavirus, for which no commercial vaccine is available [72]. Compared to the NiV-G subunit alone, this vaccine induced significantly higher levels of neutralizing antibodies, providing complete protection against lethal NiV challenges in Syrian hamsters. These findings may pave the way for more ferritin-based vaccines targeting paramyxoviruses, including avian NDV and aquatic viruses such as cetacean morbillivirus (CeMV), which infects marine mammals, as well as Pacific and Atlantic salmon paramyxoviruses

### 3.7. Infectious Hematopoietic Necrosis Virus (IHNV)

Viral diseases present a significant challenge in aquaculture, due to the lack of antiviral therapeutics and the difficulty in developing effective and safe vaccines that can be mass-administered during the early life stages of the fish, when susceptibility to viral infections is particularly high [76,77,78]. While subunit vaccines are generally safer and more cost-effective than conventional methods, their efficacy can vary. Also, these vaccines are administered by IP injection, which can be stressful and challenging for small fish. Nevertheless, the development of oral subunit vaccines has been hampered by issues such as instability of the recombinant proteins and the gastrointestinal barriers [77,79].

A promising strategy to enhance the stability and immunogenicity of fish subunit vaccines is to display structurally defined antigenic proteins in high copy numbers on the surfaces of self-assembling nanocages like ferritin.

We have recently evaluated the ferritin platform for the first time for a non-mammalian pathogen, Infectious Hematopoietic Necrosis Virus (IHNV) [10], an enveloped rhabdovirus notifiable to the WOAH that causes major epizootics in aquaculture, with mortality rates of up to 100% in small fish worldwide [80]. Currently, there are no therapeutic treatments or globally available commercial vaccines for IHNV.

Our results demonstrated the antiviral activity of the vaccine NPs, likely through the type I interferon-mediated Jak/STAT signaling pathway. Even at a lower dose of 25 µg/mL, the vaccine upregulated the expression of IHNV infection-related gene markers (mx, vig1, ifit5, and isg-15) in trout macrophages [10]. For oral delivery purposes, they investigated the stability of ferritin and the IHNV–Ferritin Vaccine under various storage, pH, and temperature conditions that mimic the gastrointestinal environment of the virus-host fish. The results demonstrated excellent stability of the NPs. This study indicates significant commercial potential for this approach in controlling fish viruses, and provides initial data on the ferritin platform for developing more aquaculture viral vaccines with sufficient immunogenicity and stability for mass administration via oral delivery to large numbers of highly susceptible small fish.

### 3.8. Other Zoonotic Viruses

The ferritin platform has also been evaluated for human use against several zoonotic viruses with enveloped RNA genomes, for which animals serve as reservoirs. Rong et al. [21] constructed a ferritin-based Zika vaccine via envelope protein domain III conjugation, which provided complete protection against viral infection and eliminated pathological symptoms in the brains of challenged mice. Ferritin nanoparticles displaying either wild-type or hyperglycosylated glycoproteins induced strong neutralizing antibodies against the Ebola virus in mice, with cross-neutralizing effects against Bundibugyo and Sudan viruses. This approach offers a potential strategy for developing universal Ebola vaccines with broad protection against various filoviruses [73].

Middle East respiratory syndrome coronavirus (MERS-CoV) is another zoonotic pathogen, transmitted to humans from dromedary camels. Kim et al. [51] engineered a fusion of the MERS-CoV RBD with *H. pylori* ferritin and an RNA-interaction domain, resulting in soluble nanoparticles produced in an *E. coli* expression system. Antibodies produced by the vaccinated mice effectively inhibited the binding of MERS-CoV RBD to the cellular receptor hDPP4. This study may have laid the groundwork for much of the extensive research conducted on COVID-19 vaccines during the pandemic [16,35,49]. Overall, these results position the ferritin platform as an ideal vaccine design strategy with commercial potential against infectious viruses. This approach may also be considered for more veterinary vaccines and intervention strategies targeting reservoir animals, aligning with the One Health approach (co-development of human and animal vaccines).

## 4. Challenges and Future Perspectives

Self-assembling ferritin nanocages represent a novel platform for vaccine candidates against various viruses, some of which have already entered human clinical trials. Experimental evidence supports the potential of this platform to develop commercial vaccines against animal and zoonotic viruses. There is growing evidence that ferritin-based vaccines can induce robust cellular and humoral immune responses comparable to those elicited by commercial whole-cell vaccines. They are safe, exhibit DIVA characteristics, are highly stable, require less-stringent cold chain logistics for transport and storage, and allow for oral administration. The flexibility of platform allows for the development of combination vaccines that target multiple diseases in a single shot. The genetic fusion of short antigen fragments to the platform also enables the rescue and production of soluble proteins that are otherwise difficult to obtain through conventional methods. Mass production of these vaccines using a low-cost expression system (*E. coli*) has further streamlined manufacturing processes.

Although several ferritin-based vaccines are currently in clinical trials, their field feasibility is still being evaluated, and there are several considerations regarding their practical use. The production of ferritin-based vaccines can be technically challenging. It requires precise engineering to ensure correct antigen display and stability. Ensuring the effective homogenization of ferritin vaccines involves carefully optimizing the antigen attachment to the ferritin platform. The expression and purification of the recombinant protein NPs must also be customized for each potential vaccine.

Moreover, ferritin is most effective for small to medium-sized antigens. Larger proteins or complex antigens using genetic fusing and chemical conjugations may not fit well within the ferritin structure, limiting its applicability for certain pathogens. To date, most ferritin-based vaccine candidates target enveloped RNA viruses, with the exception of FMDV, which is a non-enveloped virus and has provided only partial protection. This suggests that ferritin, in place of lipid membranes and matrix proteins, serves as an ideal scaffold for enveloped viruses and may be more suitable for these viruses than for non-enveloped structures.

As a novel vaccine platform, ferritin-based vaccines are subject to rigorous regulatory scrutiny. Extensive clinical trials are essential to establish their safety and efficacy, and navigating the regulatory landscape can be both time-consuming and complex. Despite these challenges, ongoing research and advancements in vaccine technology continue to address these issues, potentially enhancing the feasibility and effectiveness of ferritin-based vaccines in the future.

Different protection reports for livestock and zoonotic viruses, along with the constant emergence of new strains, highlight the need for cross-protection. Therefore, in addition to gaining a deeper understanding of the underlying immune mechanisms, the effects of various adjuvants should be investigated. Furthermore, alternative administration routes, such as stress-free oral delivery, should be explored.

A significant challenge in aquaculture is the development of effective and safe vaccines that can be mass-administered to young fish, especially during their early life stages when they are highly susceptible to viral infections. Recently, we demonstrated the stability of ferritin-based vaccines for oral administration with strong antiviral effects against the WOAH-listed IHNV fish virus, suggesting the suitability and commercial potential of the platform for developing more viral vaccines in this industry. However, in silico design of such vaccines is hindered by the lack of epitope prediction programs specifically for fish.

The ferritin platform also holds promise for developing safer and more effective vaccines for the poultry industry, where conventional vaccines are still widely used, despite challenges posed by both existing and emerging infectious diseases.

Overall, the development of ferritin-based vaccines shows tremendous promise for addressing a wide range of animal and zoonotic viruses, thereby overcoming the limitations of currently available commercial vaccines. This platform can also be rapidly deployed in response to emerging epidemics or pandemics, making it a valuable strategy for advancing veterinary vaccine development.

## Data Availability

No new data were created or analyzed in this study. Data sharing is not applicable to this article.
